# Methylprednisolone alleviates multiple sclerosis by expanding myeloid‐derived suppressor cells via glucocorticoid receptor β and S100A8/9 up‐regulation

**DOI:** 10.1111/jcmm.15928

**Published:** 2020-10-23

**Authors:** Zhongkun Wang, Ge Zheng, Guangjian Li, Mengkun Wang, Zhanchuan Ma, Huimin Li, Xiang‐Yang Wang, Huanfa Yi

**Affiliations:** ^1^ Central Laboratory The First Hospital of Jilin University Changchun China; ^2^ Key Laboratory of Organ Regeneration and Transplantation Ministry of Education Changchun China; ^3^ Vasculocardiology Department The Second Hospital of Jilin University Changchun China; ^4^ Hepatopancreatobiliary Surgery Department The Second Hospital of Jilin University Changchun China; ^5^ Neurology Department The First Hospital of Jilin University Changchun China; ^6^ Pediatric Department The First Hospital of Jilin University Changchun China; ^7^ Clinical Laboratory The Second Hospital of Jilin University Changchun China; ^8^ Department of Human and Molecular Genetics Virginia Commonwealth University Richmond VA USA; ^9^ Massey Cancer Center Virginia Commonwealth University Richmond VA USA

**Keywords:** autoimmune diseases, glucocorticoid receptor β, methylprednisolone, methylprednisolone pulse therapy, multiple sclerosis, myeloid‐derived suppressor cells

## Abstract

Methylprednisolone is an effective drug in the treatment of autoimmune disease, such as multiple sclerosis (MS), due to long‐acting anti‐inflammatory, antiallergic and immunosuppressant. Previous studies have noted the importance of myeloid‐derived suppressor cells (MDSC) in MS progression. However, it is still not known whether methylprednisolone could influence the ratio and function of MDSC during MS treatment. In the current study, we found an increased ratio of MDSC at the onset of EAE in mice model; but methylprednisolone pulse therapy (MPPT) did not alter the percentage and suppressive function of MDSC during disease attenuation. However, the percentage of G‐MDSC in PBMC significantly increased in patients with MS. Surprisingly, relapsing MS patients showed a significant increase in both M‐MDSC and G‐MDSC after MPPT. The disease remission positively correlated expansion of MDSC and expression of arginase‐1. Additionally, MPPT reduced the expression of inhibitory glucocorticoid (GCs) receptor β subunit on MDSC while elevating serum levels of immune regulatory S100A8/A9 heterodimer. Thus, MDSC dynamics and function in mouse EAE differ from those in human MS during MPPT. Our study suggested that GCs treatment may help relieve the acute phase of MS by expanding MDSC through up‐regulating of GR signalling and S100A8/A9 heterodimers.

## 1 INTRODUCTION

Methylprednisolone is a potent glucocorticosteroid with extensive therapeutic effect in inflammatory and autoimmune diseases treatment. It can effectively relieve ulcerative colitis, allergies, arthritis, lupus and multiple sclerosis (MS) due to immunosuppressive effects.^1^ A considerable amount of literature has been demonstrated the low incidence of side effects of methylprednisolone.^1^, ^2^ In MS patients, intravenous methylprednisolone treatment modulates gene expression profiles of CD4^+^ T lymphocytes, it can also induce Treg cells expansion and reduce proinflammatory cytokine secretion, and thus alleviates MS.^3^ However, data about the molecular mechanisms of methylprednisolone in MS treatment still need further research.

Multiple sclerosis is a complex inflammatory autoimmune disease with unknown aetiology. It is established that the pathogenesis of this disease is mainly attributed to abnormally activated T cells triggering an immune response against myelin or myelin‐forming cells (ie oligodendrocytes) in the central nervous system (CNS). Inflammatory cytokines produced by autoreactive T cells that penetrate the CNS after crossing the blood‐brain barrier cause damage to the myelin and surrounding tissues.^4^ Given the inflammatory nature associated with acute MS lesions, glucocorticoids (GCs) have been used for clinical management of MS. Methylprednisolone pulse therapy (MPPT) are considered in case of moderate functional severity or relapse.^5^, ^6^ However, these treatments are often associated with significant side effects. As no drugs are currently available for specific and effective MS control, more studies are needed to better understand the underlying basis of the disease pathogenesis, which may lead to new therapeutic opportunities.

Recent studies have demonstrated that a heterogeneous population of myeloid cells, termed myeloid‐derived suppressor cells (MDSC), mainly includes immature granulocytes and monocytes cells, play a critical role in shaping T‐cell responses in a variety of disease states.^7^ MDSC‐mediated immune suppression involves multiple mechanisms, including depletion of L‐arginine (a key nutritional factor needed for T‐cell proliferation) via arginase‐1 (Arg‐1), increased production of nitric oxide (NO) or reactive oxygen species (ROS) by inducible NO synthase (iNOS) or the NADPH oxidase (NOX), respectively.^7^ With their inherent T‐cell suppressive activity, MDSC were reported to alleviate the pathology of EAE,^8^, ^9^ a prime mouse model widely used to study MS resulting in development of several currently approved MS therapies.^10^ However, our recent work revealed that MDSC exacerbate disease severity by promoting Th17 cells in EAE and systemic lupus erythematosus (SLE), another autoimmune disease.^11^, ^12^ Given that the immunomodulatory function of MDSC in MS has yet been examined so far, these discrepancies raise an intriguing question about the role of MDSC in MS including disease severity and relapse.

In this study, we investigate the effect of MPPT on MDSC in EAE and, for the first time, we compare their functional differences of MDSC between EAE and MS patients during the MPPT treatment. We also define the molecular mechanisms underlying MDSC activity in MS patients, which may help guide the development of new therapeutic targets for treatment of MS.

## 2 MATERIALS AND METHODS

### 2.1 EAE induction and treatment

C57BL/6 mice were immunized with 50 μg myelin oligodendrocyte glycoprotein peptide 35‐55 (MOG35‐55) in CFA and treated twice with 400 ng pertussis toxin in total as described.^11^ Animals were weighed daily and scored for clinical signs of the disease on a scale from 0 to 5 depending on severity. Treatment with methylprednisolone (Pfizer Manufacturing Belgium NV) was performed by intravenous (iv) injection on three consecutive days at a dose of 100 mg/kg, starting once the mice had reached an average clinical score of 1 score.^13^ The dose was halved every 3 days gradually (namely 100 mg/kg over 3 days, 50 mg/kg over 3 days, 25 mg/kg over 3 days), and the injection was stopped on the 10th day. Control mice were injected with equal volumes of PBS. Mice were killed after administration of isoflurane for inducing inhalational anaesthesia. All animal experiments were conducted in accordance with the recommendations of Research Animal Care of the First Hospital of Jilin University and were approved by the Subcommittee on Research Animal Care of the First Hospital of Jilin University.

### 2.2 Study design

Thirty patients fulfilling the 2005 The McDonald criteria for multiple sclerosis (MS) were randomly recruited between September 2014 and December 2017 from the First Hospital of Jilin University. The disease subtypes of these patients were all relapsing‐remitting MS, characterized by an initial episode of neurological dysfunction (clinically isolated syndrome), followed by a remission period of clinical recovery and then recurring bouts of relapse and remission. Healthy volunteers were randomly recruited as healthy controls (HCs) with an effort to match age and gender of MS patients. For all experiments using clinical samples, blinded outcome assessment was conducted. All participants were provided written informed consent, and all procedures were approved by the ethics committee of the First Hospital of Jilin University. Blood samples (heparin anticoagulation) of relapsing‐RRMS patients were obtained by venipuncture before MPPT. Twelve patients received iv short‐term high‐dose MPPT. Typically, methylprednisolone iv was administered in the morning (1000 mg over 3 days, 500 mg over 3 days, 250 mg over 3 days, 125 mg over 3 days). The status of all MS patients before and after treatment was assessed. Clinical improvement evidenced by alleviations of clinical symptom indicates a therapeutic response. Blood samples were also collected immediately after the ninth GC infusion (the third administration of 250 mg methylprednisolone). The assessment of responses to GC therapy was made by physicians not involved in this study.

### 2.3 Cell preparations

PBMC were prepared by density gradient centrifugation using Histopaque^®^‐1083(Sigma, 10831) and Histopaque‐1077 (Sigma‐Aldrich). Naive CD4^+^ T cells were isolated from PBMC using the naive CD4^+^ T cell isolation kit II (Miltenyi Biotec) according to the manufacturer's instructions, and the purity of cells after separation was >98%. MDSC were isolated from PBMC by cell sorting using a cell sorter (Influx; Becton Dickinson).

### 2.4 Suppression of T‐cell proliferation

Control splenocytes (2 × 10^5^ cells/well) were cocultured with purified MDSC at 1:1 ratio in the presence of 2 µg/mL plate‐bound anti‐CD3 mAb (be0001‐1; BioXcell) and 1 μg/mL soluble anti‐CD28 mAb (102112; BioLegend) in a 96‐well flat‐bottom plate for 72 hours. Naïve human CD4^+^ T cells(3 × 10^5^ cells/well) with purified MDSC at 1:1 ratio in the presence of 10 µg/mL plate‐bound anti‐human CD3 (OKT‐3; BioXcell) and 5 μg/mL soluble anti‐human CD28 mAb (CD28.2; eBioscience) in a 96‐well flat‐bottom plate for 5 days. Subsequently, T‐cell proliferation was determined by measuring BrdU incorporation (BrdU was added 6 hours prior to cell harvest) (Roche).

### 2.5 Flow cytometric analysis

Flow cytometry was used to determine the phenotypes of mouse MDSC, human MDSC, and Treg cells using various combinations of the following fluorochrome‐conjugated mAbs: anti‐mouse CD11b (101226; BioLegend), anti‐mouse Gr‐1 (108428; BioLegend), anti‐mouse Ly6C(25‐5932‐82; eBioscience), anti‐mouse Ly6G(11‐5931‐82; eBioscience), anti‐human HLA‐DR (555561; BD), CD11b (101226; BioLegend), CD33 (303416; BioLegend), CD14 (301804; BioLegend), CD66b (305108; BioLegend); anti‐human arginase‐1 (IC8026G; R&D Systems); CD14 (301842; BioLegend); Isotype controls mouse IgG2b (IC0041G; R&D Systems); Anti‐Glucocorticoid Receptor alpha antibody (ab3580; Abcam), Anti‐Glucocorticoid Receptor beta antibody (ab130227 and ab3581; Abcam), Anti‐Glucocorticoid Receptor antibody (ab109022; Abcam), Goat Anti‐Rabbit IgG H&L (Alexa Fluor^®^ 488) (ab150077; Abcam), and Goat Anti‐Mouse IgG H&L (Alexa Fluor^®^ 488) (ab150113; Abcam). Isotype controls used Rabbit IgG, polyclonal (ab171870; Abcam), Rabbit IgG, monoclonal (ab199376; Abcam), and Mouse monoclonal IgG1(ab170190; Abcam) from Abcam. For intracellular staining, cells were first stained for surface antigens, fixed, and permeabilized with intracellular fixation and permeabilization buffer (eBioscience). Next, the samples were stained with fluorochrome‐conjugated mAbs against their respective intracellular proteins. All samples were collected on a fluorescence‐activated cell sorter (LSRFortessa; Becton Dickinson) and analysed by FlowJo software (Tree Star).

### 2.6 Quantification of serum arginase activity, iNOS and S100A8/A9

Arginase activity was quantified by the QuantiChrom arginase assay kit (Bioassay Systems) according to the manufacturer's instructions and data expressed as enzyme activity (units per litre). Serum levels of human iNOS were measured using an ELISA kit according to the manufacturer's instructions. Serum levels of S100A8/A9 were measured using an ELISA kit according to the manufacturer's instructions (439707; BioLegend).

### 2.7 Quantitative real‐time PCR

RNA was extracted, and complementary DNA was synthesized using the PrimeScript RT reagent kit with gDNA Eraser (Perfect Real Time) (RR047; TaKaRa). All PCRs were performed in triplicate and carried out on an ABI StepOnePlus system (Applied Biosystems) with TB Green^™^ Premix Ex Taq^™^ (Tli RNaseH Plus) (RR420; TaKaRa). mRNA expression levels were quantified using primers for harginase‐1 (DHS006400, XY Biotech, *hiNOS* (DHS226672; XY Biotech), *hIDO* (DHS606951; XY Biotech), *hATF3* (DHS013101; XY Biotech), *hS100A8* (DHS479724; XY Biotech), *hS100A9* (DHS767566; XY Biotech), and actin was used as previously described.^14^ The other primer sequences are as follows: *hIRF7*, 5′‐CCCACGCTATACCCATCTACCT‐3′ (forward) and 5′‐GATGTCGTCATAGAGGCTGTTG‐3′ (reverse); and *hmactin*, 5′‐TTCAACACCCCAGCCATG‐3′ (forward) and 5′‐CCTCGTAGATGGGCACAGT‐3′ (reverse).

### 2.8 Statistical analysis

Statistical analyses were performed on GraphPad Prism 5.0 software. Data are expressed as means ± SD. Between‐group comparisons were performed using two‐tailed *t* test, whereas multiple‐group comparisons were performed using one‐way analysis of variance (ANOVA) followed by the Newman‐Keuls test. The Spearman rank test was used for analysis of correlation. A *P* value of <0.05 was considered significant.

## 3 RESULTS

### 3.1 The expansion of MDSC was independent of GC treatment in EAE

**FIGURE 1:**
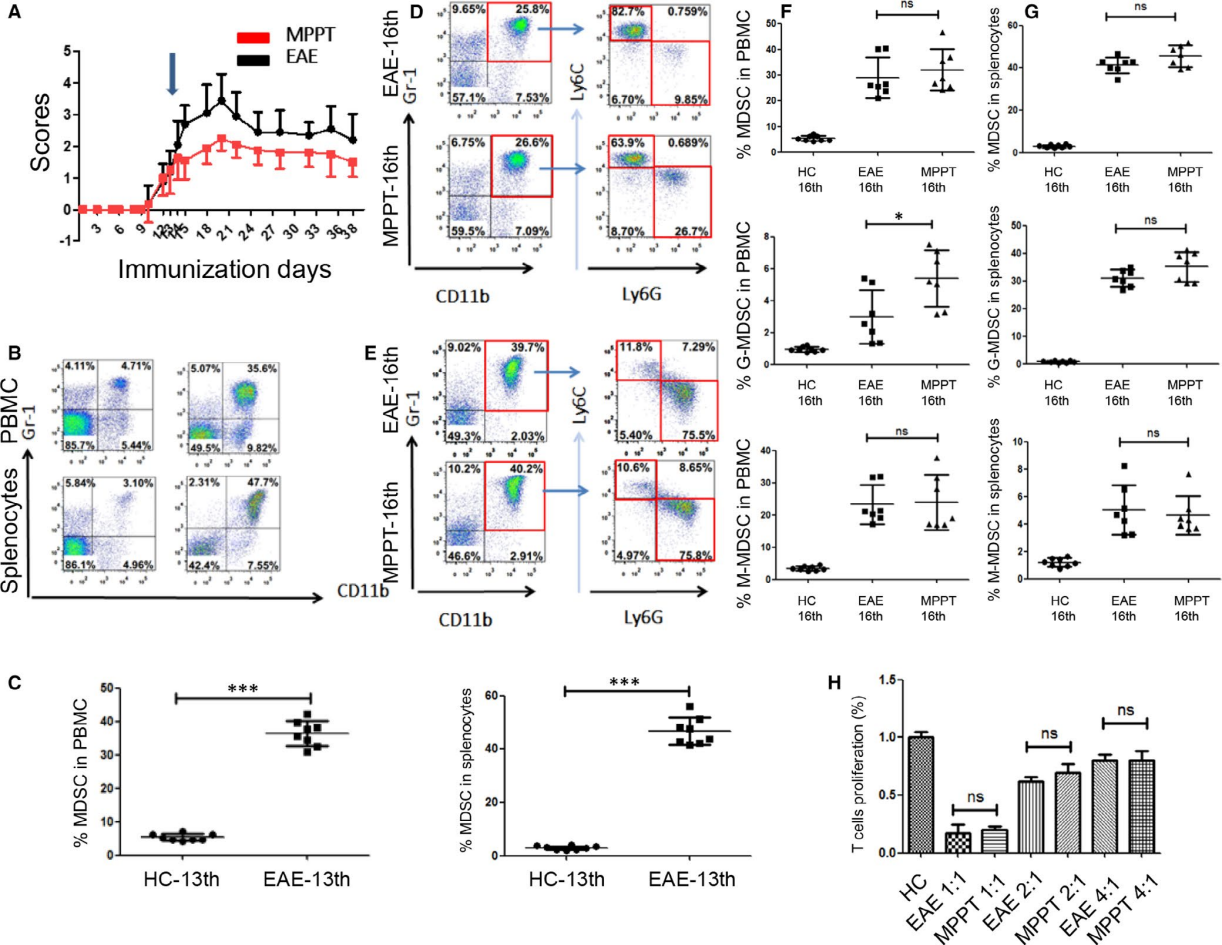
The expansion of MDSC is independent of GC treatment in EAE. A, EAE models were successfully established. Methylprednisolone (100 mg/kg) was administered after EAE induction on the 13th day (blue arrow, average clinical score = 1). The dose was halved every 3 d gradually (100 mg/kg for 3 d, 50 mg/kg for 3 d, 25 mg/kg for 3 d). The injection was stopped on the 10th day after the initial injection. EAE development in mice (each group n = 10) was followed, and clinical scores were recorded (*P* < 0.001, MPPT vs EAE). B, Representative staining profiles of MDSC from PBMC from the healthy control (HC) and EAE groups. The cells were collected on the 13th day. C, Percentages of MDSC from PBMC (*P* < 0.0001) and whole splenocyte population (*P* < 0.0001) of the EAE and MPPT groups. The cells were collected on the 13th day. D‐E, Representative staining profiles of MDSC, M‐MDSC, and G‐MDSC from whole splenocyte populations from the EAE and MPPT groups. The cells were collected on the 16th day. F, Percentages of MDSC (*P* = 0.4973), G‐MDSC (*P* = 0.0231, *t* test), M‐MDSC *(P*=0.8649) from PBMC of the EAE and MPPT groups. The cells were collected on the 16th day. G, Percentages of MDSC (*P* = 0.0947), G‐MDSC *(P*=0.1037), M‐MDSC (*P* = 0.6652) from the whole splenocyte population of the EAE and MPPT groups. The cells were collected on the 16th day. H, Suppressive function of MDSC and their subsets from splenocytes were evaluated between the EAE and MPPT groups on the 16th day. *P < 0.05; ***P < 0.001

Our previous work showed that MDSC in PBMC and spleen significantly increased at the early stage of EAE and contributed to the pathogenesis of EAE ^11^ (Figure 1B‐C). To clarify the effect of MPPT on MDSC in EAE, we injected Methylprednisolone intravenously to EAE mice when the score reached average 1(Figure 1A). Then, we found that GC did not significantly change the levels of MDSC or the proportion of the two cell subsets (ie., M‐MDSC and G‐MDSC) in PBMC and spleen, although MPPT alleviated the clinical symptoms of EAE as expected (Figure 1D‐G). Only the percentage of G‐MDSC was up‐regulated modestly after MPPT in PBMC, not M‐MDSC (Figure 1F). Additionally, GC treatment did not alter T‐cell suppressive activity of either total MDSC or the MDSC subsets (Figure 1H). Consistent with our previous work, M‐MDSC appeared to be more immunosuppressive than G‐MDSC from mice with EAE (Figure S1).

### 3.2 Increased G‐MDSC in RRMS patients

**FIGURE 2:**
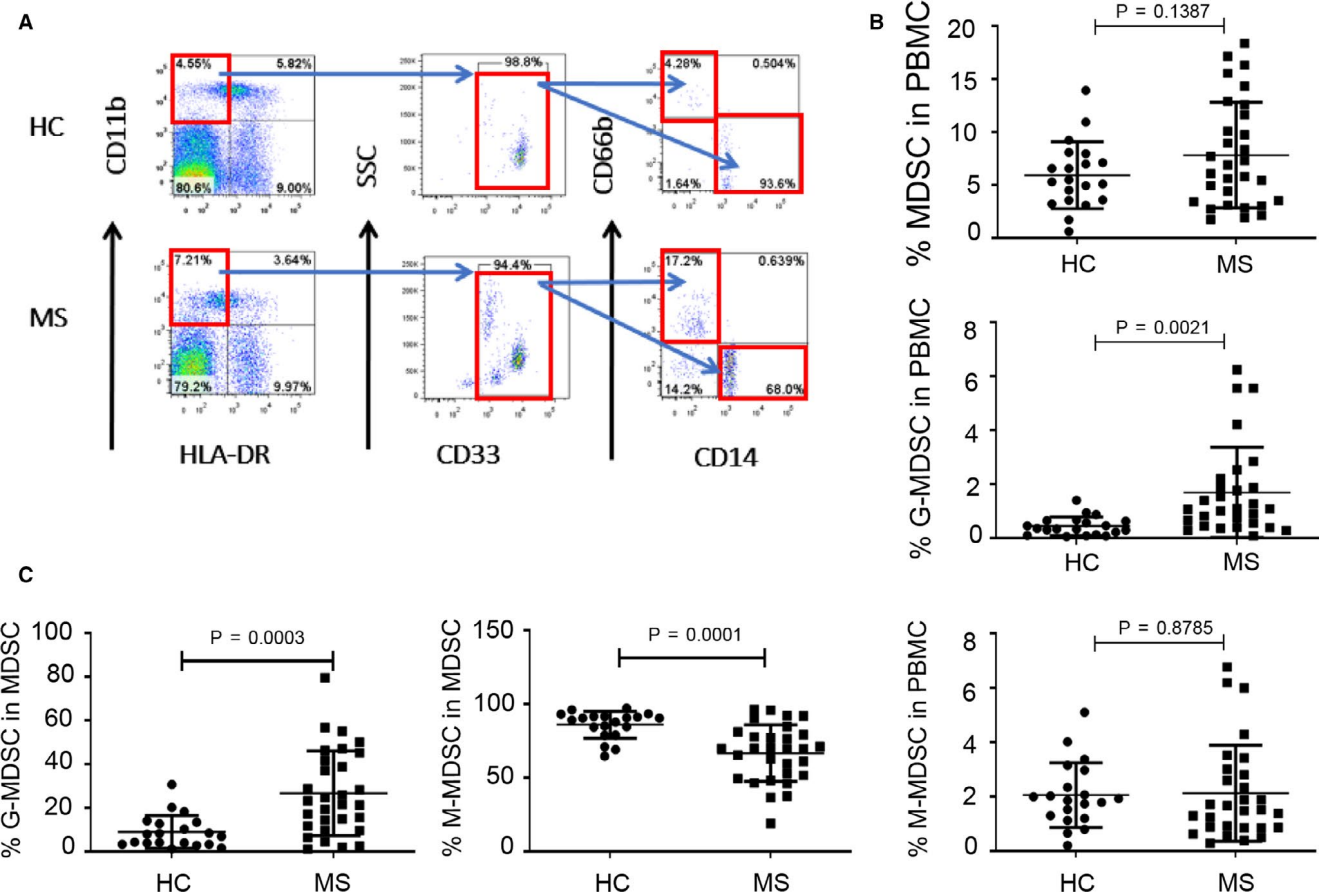
The percentages of G‐MDSC in PBMC from relapsing MS patients were significantly higher than those from HCs. A, Staining profiles of MDSC (CD11b^+^CD33^+^HLA^‐^DR^−^), M‐MDSC (CD11b^+^CD33^+^HLA^‐^DR^−^CD14^+^CD66b^−^), and G‐MDSC (CD11b^+^CD33^+^HLA^‐^DR^−^CD14^−^CD66b^+^) from a representative HC and a representative MS patient. B, Percentages of MDSC (*P* = 0.1387), G‐MDSC (*P* = 0.0021), and M‐MDSC (*P* = 0.8785) in PBMC of HCs (n = 20) and MS patients (n = 30). C, Percentages of G‐MDSC (*P* = 0.0003), and M‐MDSC (*P* = 0.0001) in MDSC of PBMC from HCs (n = 20) and MS patients (n = 30)

We next examined the frequency of human MDSC and their subsets in PBMC from patients with RRMS. MDSC were defined as CD11b^+^CD33^+^HLA‐DR^−^, which were further divided into SSC^low^CD14^+^CD66b^−^ M‐MDSC and SSC^high^CD14^−^CD66b^+^ G‐MDSC subsets (Figure 2A). The percentages of MDSC or M‐MDSC subset in PBMC from RRMS patients did not significantly change compared with those in HCs (Figure 2A,B). However, the PBMC from RRMS patients showed a significant increase in G‐MDSC (Figure 2A,B). Interestingly, while the percentage of G‐MDSC in total MDSC increased that of M‐MDSC in total MDSC decreased (Figure 2C).

### 3.3 Alleviation of MS by GC correlated with expansion of G‐MDSC

**FIGURE 3:**
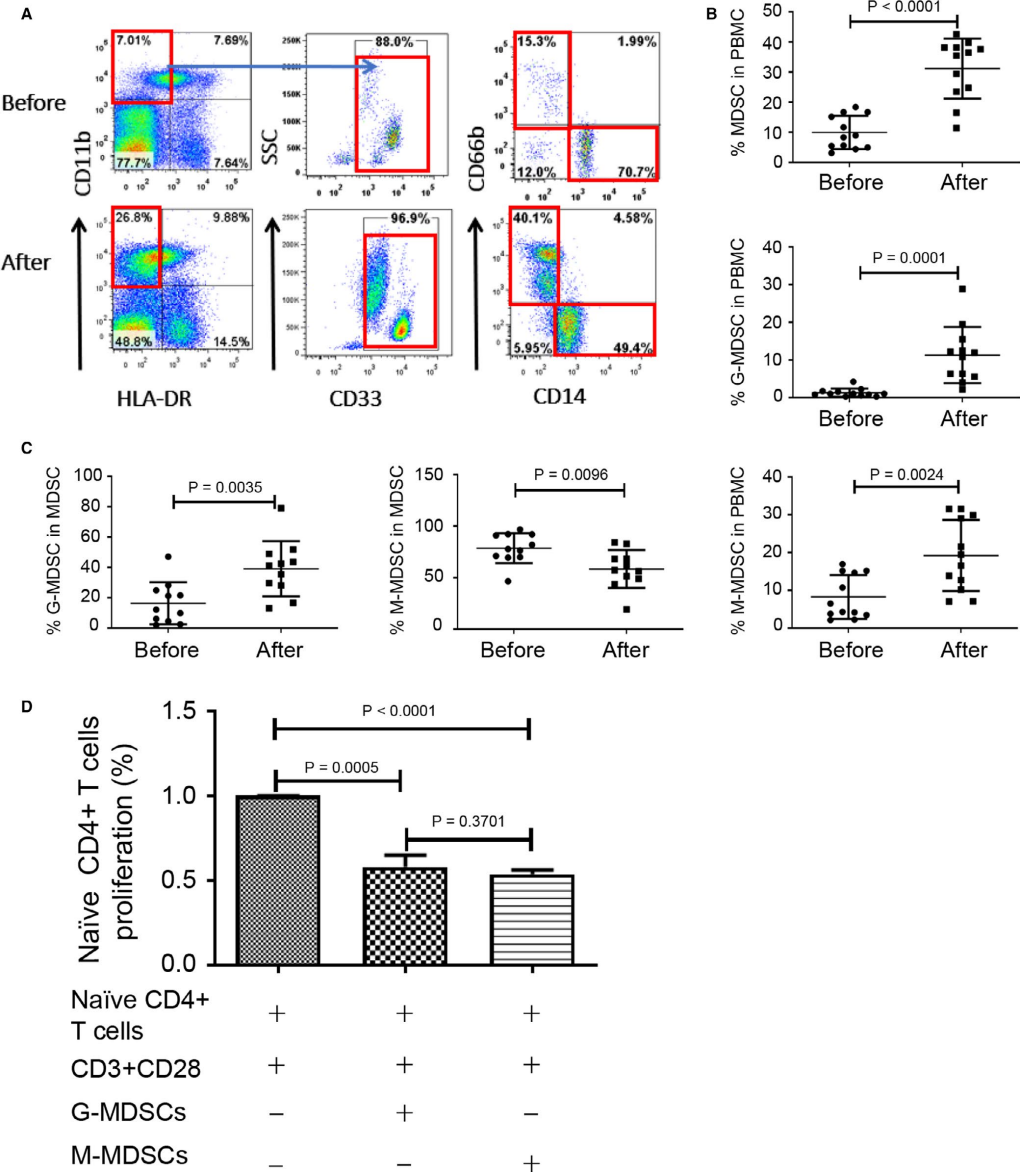
Glucocorticoid treatment amplified the percentages of MDSC and the two subsets in PBMC of relapsing MS patients. A, Staining profiles of MDSC (CD11b^+^CD33^+^HLA^‐^DR^−^), M‐MDSC (CD11b^+^CD33^+^HLA^‐^DR^−^CD14^+^CD66b^−^) and G‐MDSC (CD11b^+^CD33^+^HLA^‐^DR^−^CD14^−^CD66b^+^) from a representative MS patient before and after methylprednisolone impulse treatment. B, Percentages of MDSC (*P* < 0.0001), G‐MDSC (*P* = 0.0001) and M‐MDSC (*P* = 0.0024) in PBMC of MS patients (n = 12) before and after treatment. C, Percentages of G‐MDSC (*P* = 0.0035) and M‐MDSC (*P* = 0.0096) in MDSC of PBMC from MS patients (n = 12) before and after treatment. D, Naïve human CD4^+^ T cells were stimulated by anti‐CD3/CD28 in the absence or presence of MDSC from MS patients for 3 d (G‐MDSC, *P* = 0.0005, n = 3; M‐MDSC, *P* < 0.0001, n = 3; G‐MDSC vs M‐MDSC, *P* = 0.3701, n = 3), and T‐cell proliferation was determined by measuring BrdU incorporation (Brdu was added 6 h prior to cell harvest)

To determine an impact of GC on MDSC in MS patients, we assessed the changes of MDSC before and after MPPT. We showed that percentages of MDSC in PBMC elevated significantly after GC treatment (Figure 3A,B). Among these two cell populations, only the G‐MDSC significantly elevated but not M‐MDSC (Figure 3B,C). Additionally, MDSC from MS patients after MPPT inhibited T‐cell proliferation (Figure 3D), suggesting that these cells retain a T‐cell suppressive activity. These results indicated that MS patients differ from the mouse EAE model in terms of the dynamics and function of MDSC.

### 3.4 Increased serum Arg‐1 activity and Arg‐1 production by G‐MDSC

**FIGURE 4:**
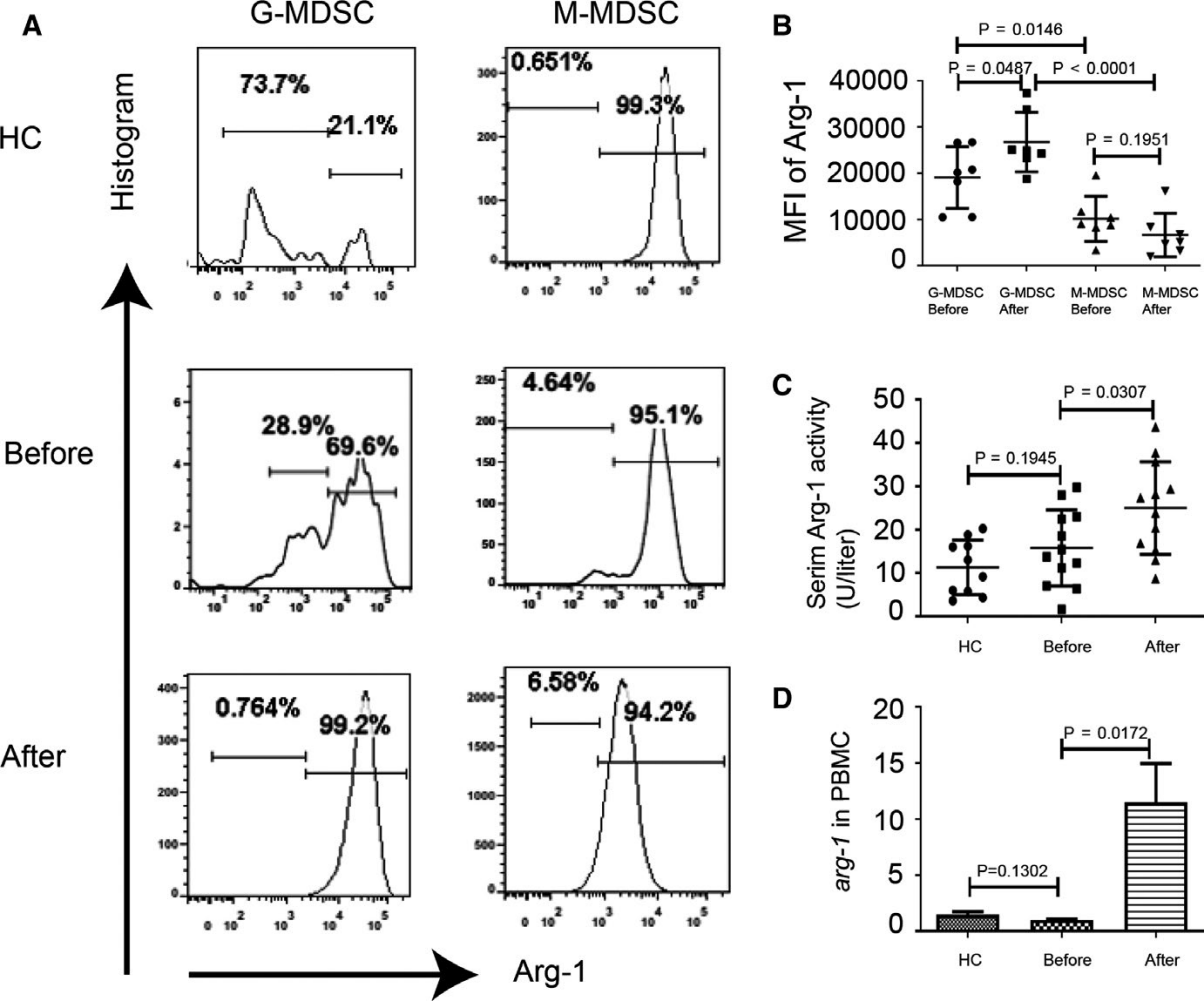
Serum Arg‐1 activity was more strongly correlated with the percentages of G‐MDSC in relapsing MS patients. A, Representative images of immunofluorescence staining for Arg‐1 in M‐MDSC and G‐MDSC from a representative HC and a representative MS patient before and after treatment. B, Levels of Arg‐1 expression in PBMC of MS patients (mean fluorescence intensity; means ± SD; n = 7 per group; (G‐MDSC before treatment vs G‐MDSC after treatment, *P* = 0.0702, M‐MDSC before treatment vs M‐MDSC after treatment, *P* = 0.1951, G‐MDSC vs M‐MDSC before treatment, *P* = 0.0146, G‐MDSC vs M‐MDSC after treatment, *P* < 0.0001) (C) Arginase activity in sera from HCs (n = 10) and MS patients (n = 12) before and after methylprednisolone impulse treatment (HCs vs MS patients before treatment, *P* = 0.1945; MS patients before treatment vs MS patients after treatment, *P* = 0.0307). D, Levels of *Arg‐1* mRNA were measured by qPCR (HCs vs MS patients before treatment, *P* = 0.1302; MS patients before treatment vs MS patients after treatment, *P* = 0.0172, n = 5)

To better understand the molecular pathways by which MDSC, which expanded in MS patients, exerts their suppressive function, we examined several factors including Arg‐1 that is known to mediate the immunosuppressive activity of MDSC.^7^ Intracellular staining for Arg‐1 showed that G‐MDSC express much more Arg‐1 compared with M‐MDSC (Figure 4A,B) and production of Arg‐1 in G‐MDSC was further enhanced by GC treatment (Figure 4B). We next addressed the question whether GC treatment altered production of Arg‐1 by measuring serum Arg‐1 activity in MS patients before and after MPPT. There was no obvious difference in Arg‐1 activity between untreated MS patients and HCs (Figure 4C). However, a significant increase in serum Arg‐1 activity was detected in all MS patients after treatment (Figure 4C). Additionally, our results showed a marked up‐regulation of *arg‐1* mRNA after MPPT in PBMC (Figure 4D).

**FIGURE 5:**
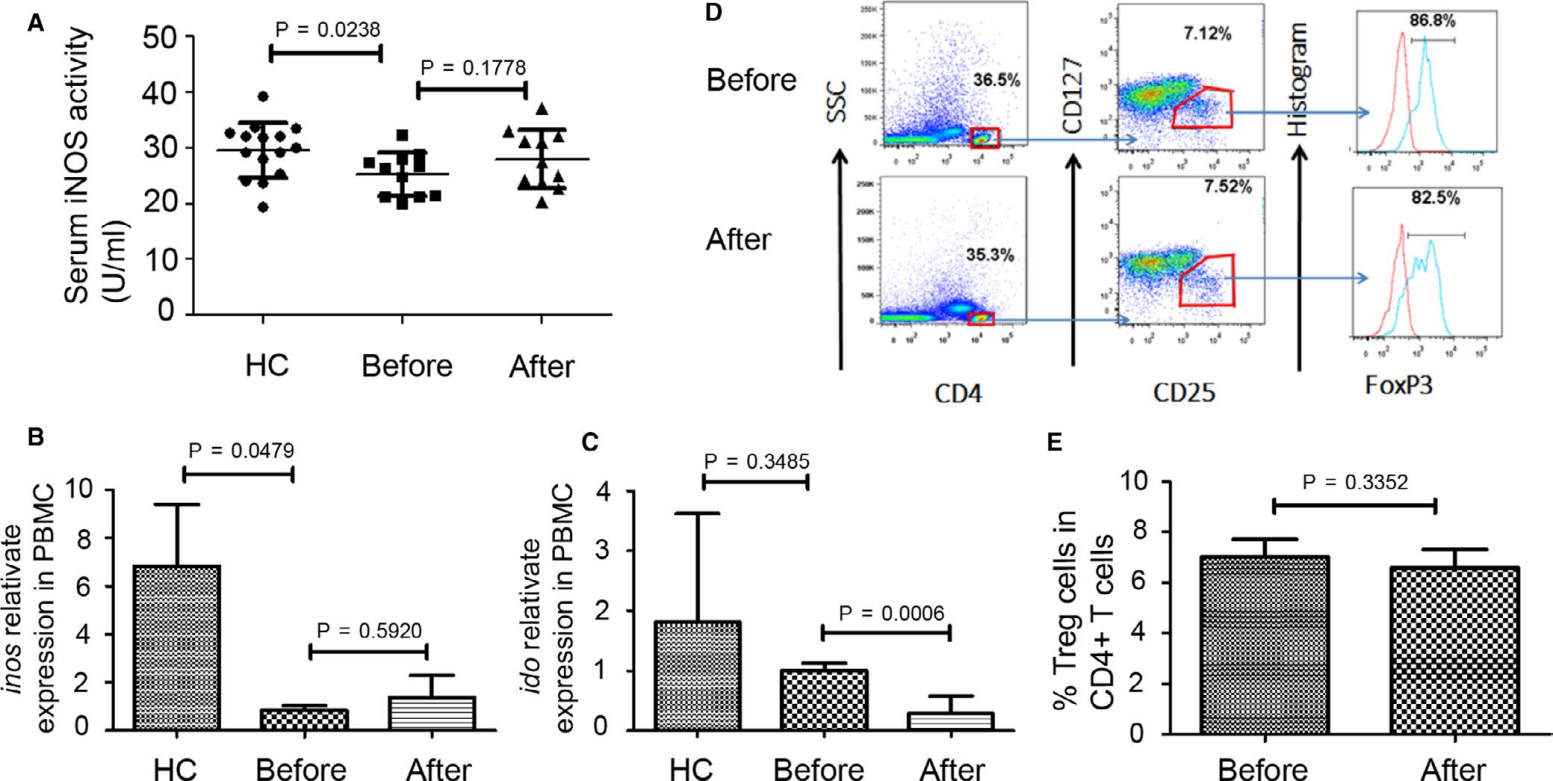
There were no significant increases in serum iNOS levels, *inos* and *ido* mRNA expression, and percentages of Treg cells in relapsing MS patients after MPPT. A, Serum iNOS activity from HCs (n = 15) and MS patients (n = 11) before and after methylprednisolone impulse treatment (HCs vs MS patients before treatment, *P* = 0.0238; MS patients before treatment vs MS patients after treatment, *P* = 0.1778). B, Levels of *inos* mRNA were measured by qPCR (HCs vs MS patients before treatment, *P* = 0.0479; MS patients before treatment vs MS patients after treatment, *P* = 0.5920, n = 5). C, Levels of *ido* mRNA were measured by qPCR (HCs vs MS patients before treatment, *P* = 0.3485; MS patients before treatment vs MS patients after treatment, *P* = 0.0006, n = 5). D, Staining profiles of CD4^+^ T cells (CD4+) and Treg cells (CD4^+^CD25^+^CD127^−^Foxp3^+^) from a representative MS patient before and after methylprednisolone impulse treatment. E, Percentages of Treg cells in CD4^+^ T cells of MS patients’ PBMC (n = 5) before and after treatment (*P* = 0.3352)

In addition to Arg‐1, several other mechanisms have been implicated in MDSC‐mediated immunosuppression, such as iNOS that is mainly produced by M‐MDSC.^7^ Examination of serum iNOS levels in MS patients and HCs showed no significant increase in serum iNOS levels in MS patients after treatment (Figure 5A). qPCR analysis of PBMC also did not detect a significant change in *iNOS* mRNA after MPPT (Figure 5B). As *IDO* is also produced by MDSC,^15^ we examined the levels of *IDO* mRNA in PBMC. Surprisingly, *IDO* mRNA expression from MS patients was reduced by MPPT (Figure 5C). MDSC have been shown to promote immune tolerance through expansion of Treg cells in mouse and human studies.^7^ However, we showed that GC did not change the percentages of CD4^+^CD25^+^CD127^−^Foxp3^+^ Treg cells in PBMC of MS patients (Figure 5D,E). Collectively, our results showed an increased Arg‐1 secretion in MDSC of MS patients after MPPT.

### 3.5 Negative correlation between expansion of MDSC and hGRβ expression

**FIGURE 6:**
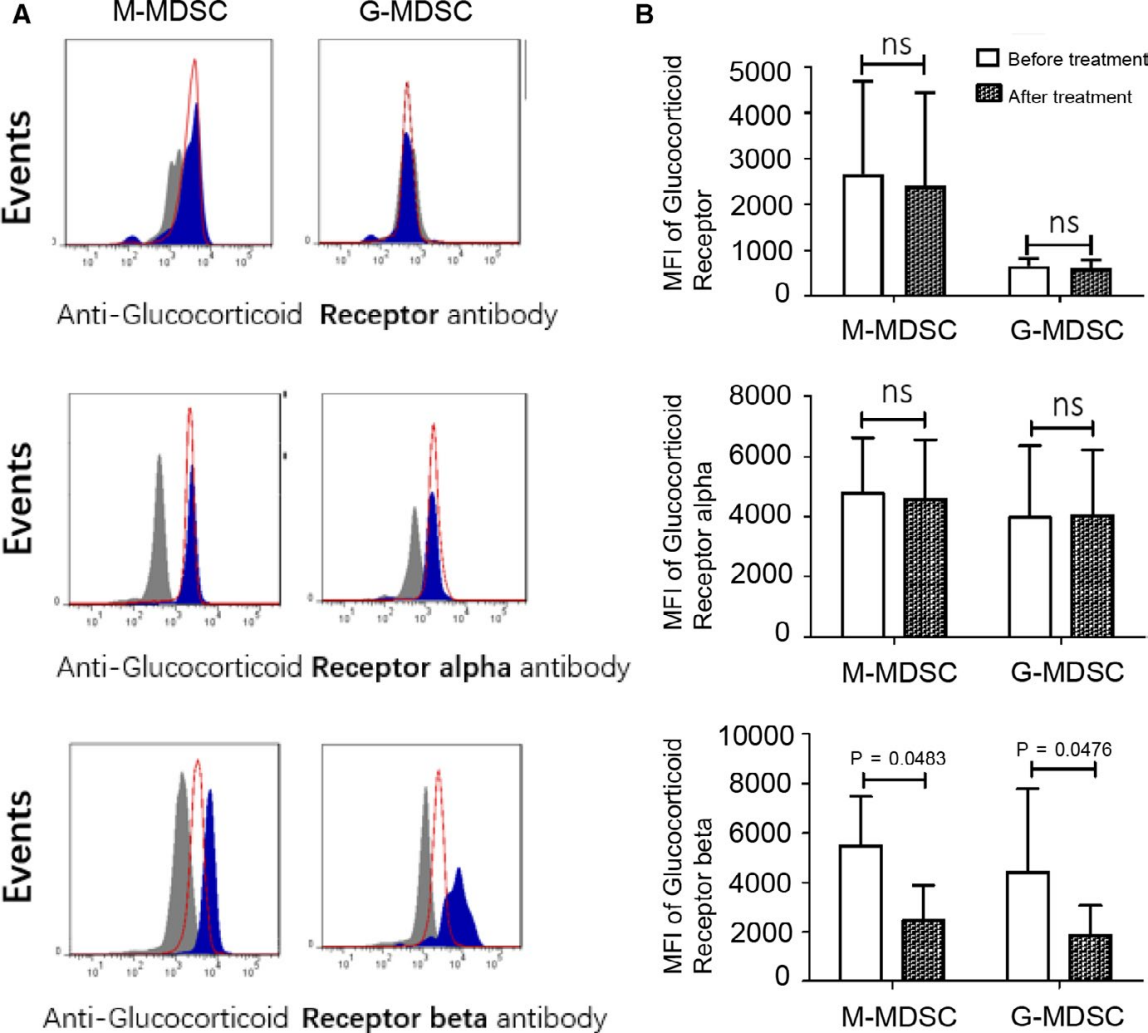
Glucocorticoid receptor expression in two MDSC subsets of relapsing MS patients before and after treatment. A, B, Glucocorticoid receptors (glucocorticoid total receptor, glucocorticoid receptor alpha and glucocorticoid receptor beta) expression in M‐MDSC (n = 4 per group; glucocorticoid total receptor *P* > 0.05; glucocorticoid receptor alpha *P* > 0.05; glucocorticoid receptor beta *P* = 0.0483) and G‐MDSC (n = 4 per group; glucocorticoid total receptor *P* > 0.05; glucocorticoid receptor alpha *P* > 0.05; glucocorticoid receptor beta *P* = 0.0476) from MS patients before MPPT and MS patients after treatment. Fig A shows staining profiles of cells from a representative MS patient before treatment (blue filled) and a representative MS patient after treatment (red line); grey filled: isotype control. Fig B shows representative results of four independent experiments

To explore how GC treatment expanded MDSC in MS patients, we assessed the expression of human glucocorticoid receptor (hGR), which has two isoforms, hGRα and hGRβ.^16^ The expression of total hGR or hGRα on MDSC subsets did not change before or after GC treatment (Figure 6A,B). However, the levels of hGRβ expression on these cells significantly decreased after GC treatment (Figure 6A,B), implicating a potential role of hGRβ for modulating MDSC amplification.

### 3.6 Association of S100A8/A9 with MDSC accumulation in MS patients

**FIGURE 7:**
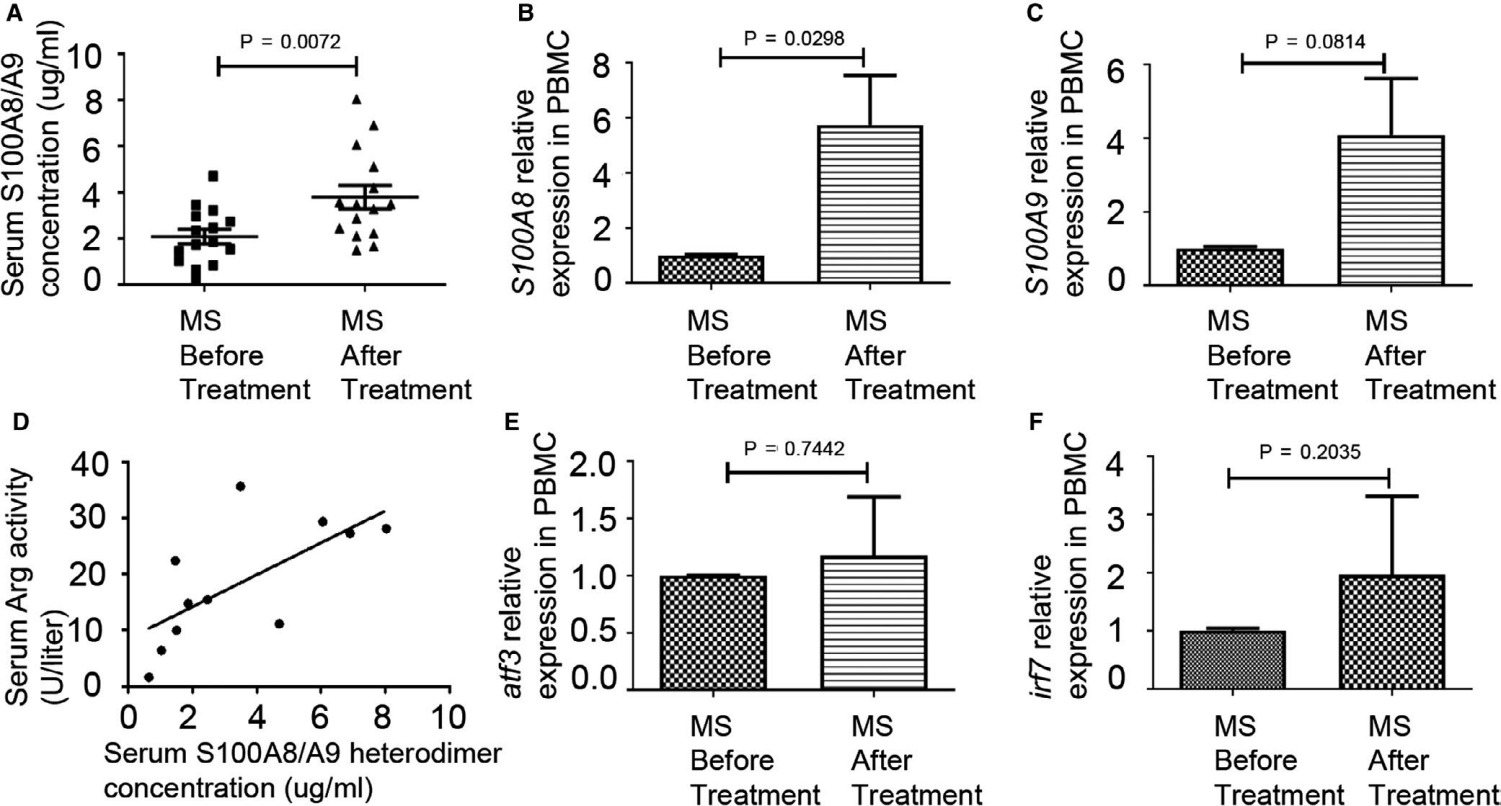
Immunoregulatory S100A8/A9 heterodimer induces the accumulation of MDSC in MS patients after glucocorticoid treatment. A, Serum S100A8/A9 heterodimer concentration in serum from MS patients (n = 15) before and after MPPT (MS patients before treatment vs MS patients after treatment, *P* = 0.0072). B, Levels of S100A8 mRNA were measured by qPCR (MS patients before treatment vs MS patients after treatment, *P* = 0.0298, n = 5). C, Levels of S100A9 mRNA were measured by qPCR (MS patients before treatment vs MS patients after treatment, *P* = 0.0814, n = 5). D, Correlation analysis between serum arginase activity and serum S100A8/A9 heterodimer concentration in MS (*r* ^2^ = 0.4563, *P* = 0.0225). E, Levels of Atf3 mRNA were measured by qPCR (MS patients before treatment vs MS patients after treatment, *P* = 0.7442, n = 5). F, Levels of Irf7 mRNA were measured by qPCR (MS patients before treatment vs MS patients after treatment, *P* = 0.2035, n = 5)

Activating transcription factor 3 (ATF3)/S100A9 signalling plays an important role in GC‐mediated G‐MDSC accumulation in the fatty liver disease.^17^ GC can directly induce A8/A9 mRNA in human monocytes or A8/A9‐positive cells in the rheumatoid synovial membrane.^18^ To examine a potential role that S100A8/A9 protein may play in GC‐induced MDSC expansion, serum samples from MS patients were assayed for the levels of S100A8/A9 heterodimer. We showed that methylprednisolone significantly increased S100A8/A9 protein levels (Figure 7A), which was associated with up‐regulation of their mRNA levels relative to untreated patients (Figure 7B‐C). We further showed that there was a positive correlation between the serum Arg‐1 activity and serum S100A8/A9 heterodimer levels in MS patients (Figure 7D). However, the mRNA expression of cyclic AMP‐dependent transcription factor 3 (*ATF3*), a negative regulator of S100A8/A9, remained unchanged (Figure 7E).

Considering that interferon regulatory factor‐7 (IRF7) regulates the development of G‐MDSC through S100A9 gene transcription in cancer ^19^ and modulates progression of EAE in mice,^20^ we also compared relative expression of *IRF7* mRNA in PBMC of MS patients before and after MPPT. However, there was no change in *IRF7* mRNA levels (Figure 7F), indicating IRF7 may not a crucial factor in regulating G‐MDSC development after MPPT.

## 4 DISCUSSION

Glucocorticoids are the most widely used drugs to inhibit immune activation during transplantation and autoimmune diseases. Glucocorticoids can induce immunoregulatory cells such as Tregs, tolerogenic dendritic cells or MDSC expansion to indirectly inhibit immune response. Previous studies showed that MPPT was effective but the side effects should be taken into account in some diseases’ treatment. The aim of our study was to investigate the influence and mechanism of MPPT to MDSC during MS treatment, and advance the understanding of MPPT in immune regulatory aspects.

Despite well documented immunosuppressive property of MDSC, these cells have been reported to either aggravate or inhibit autoimmune diseases has been under debate.^21^ In the current study, we have determined changes of MDSC in a widely used mouse model of MS (ie EAE) or MS patients undergoing MPPT. We show that the expansion of T‐cell suppressive MDSC by GC treatment, particularly G‐MDSC, may contribute to the resolution of the acute phase of MS.

It has been well established in EAE model that CD11b^+^Gr‐1^−^ MDSC increase at the onset of disease, continue to accumulate prior to disease remission, and contracted upon disease resolution.^9^, ^11^, ^22^, ^23^, ^24^, ^25^, ^26^, ^27^, ^28^ However, we show that CD11b^+^CD33^+^HLA‐DR^−^ MDSC in MS patients do not significantly change in the relapsing phase. Indeed, there is a positive correlation between the levels of circulating G‐MDSC and disease relapse in MS patients, which is consistent with a previous report.^23^, ^29^ Additionally, GC treatment appears to expand MDSC in MS patients, whereas it has little impact on MDSC in EAE, suggesting that the dynamics of MDSC in EAE model differ from that in MS patients.

Currently, methylprednisolone plays an important role in the treatment of MS, particularly in the acute phase of MS relapse. Interestingly, we demonstrate that MDSC or its two subsets in PBMC from MS patients elevate significantly after MPPT, while no change is seen in their T‐cell suppressive activity. This positive correlation between MDSC expansion and disease remission in MS patients implicates a potential role of these MDSC in shorting the relapsing phase of MS. We previously reported that SLE patients showed a significant increase in MDSC that can exacerbate the severity of the disease by augmenting Th17 differentiation.^12^ It appears that MDSC amplified by GC treatment in MS patients functionally differ from those in SLE patients, further highlighting the complexity of context‐dependent activity of MDSC in human diseases.

We have also explored the molecular basis of MDSC‐mediated T‐cell suppression in GC‐treated MS patients. It has been shown that G‐MDSC express higher levels of ROS, whereas M‐MDSC produce more iNOS, and both cell subsets express a modest level of Arg‐1.^7^ Here, we demonstrate that expression of Arg‐1 is more prominent in G‐MDSC, which show more significant expansion than M‐MDSC. In addition, GC treatment further increases Arg‐1 production in G‐MDSC, whereas iNOS and IDO levels remain unchanged, indicated that the MPPT could not enhance the expression of iNOS and IDO in MS patients. This is consistent with a previous report showing that methylprednisolone treatment did not change the concentrations of NO degradation products in the cerebrospinal fluid during the course of an MS attack.^30^ Although MDSC can suppress T‐cell function by inducing proliferation and differentiation of Treg,^7^ our results suggest that the modest increase in Treg is less likely to contribute to disease remission by GC treatment. Thus, increased Arg‐1 level may play a key role in GC‐expanded MDSC, especially G‐MDSC, when involved in immune suppressive activity, thereby alleviate the acute phase of MS. In support of our finding, an increase in G‐MDSC in pregnant MS patients was recently shown reduce the relapse of the disease.^31^, ^32^ However, additional experiments are required to determine the suppressive function of iNOS, IDO and Treg when MDSC are investigated during MPPT in MS patients in further study.

Administration of a synthetic GC dexamethasone (Dex) was previously shown to cause expansion of distinctive proinflammatory monocytes.^33^ Accumulating studies suggest that the increased GR expression is involved in this process.^34^, ^35^, ^36^, ^37^ While there is only one receptor for GC in mice, human GRs include three isoforms, that is GRα, GRβ and GRγ. GRα is believed to direct the appropriate GC signalling transduction, whereas GRβ and GRγ antagonize GC activity. Our data show that MPPT significantly reduce expression of GRβ, not GRα on MDSC, suggesting that methylprednisolone induces the expansion of MDSC or G‐MDSC by promoting GC‐GRα signalling in the acute relapse phase of MS.

S100A8 (A8, MRP8, calgranulin A) is generally co‐expressed with S100A9 (A9, MRP14, calgranulin B). Recently, it was reported that MDSC and S100A8/A9, co‐expressed members of the S100 family of calcium‐binding proteins that have an immunoregulatory role,^18^, ^38^ can operate through a positive feedback loop to promote tumour development and metastasis.^39^ For the first time, we show that methylprednisolone significantly increases S100A8/A9 protein expression that is associated with the serum Arg‐1 activity, suggesting that the immunoregulatory S100A8/A9 heterodimer may mediate GC‐caused MDSC expansion in MS patients. The transcription factor interferon Regulatory Factor‐7 is constitutively expressed at low levels in the cytoplasm ^40^ and becomes activated by innate receptor signalling, resulting in translocation to the nucleus and induction of type I IFN.^41^ Mice lacking either IFN‐β or IFNAR develop more severe EAE, with increased CNS infiltration.^42^, ^43^ Our results showed that there was no significant change in *IRF7* mRNA levels in PBMC of MS patients before and after MPPT, indicating *IRF7* may not a crucial factor in regulating G‐MDSC development after MPPT.

In summary, there is a distinction in MDSC from EAE and from MS in that these cells are not primarily involved in MPPT‐reduced disease severity in EAE model; however, they play an important role in the acute GC therapy of MS patients. Our findings indicate the pathogenic complexity of MS as well as functional diversity of MDSC during treatment. Additionally, this is the first study that reveals a novel mechanism involving expansion of immunosuppressive MDSC, particularly G‐MDSC, which is responsible for GC treatment in the acute relapse phase of MS. Furthermore, our molecular studies uncover that amplification of MDSC by GC may be attributed to GR signalling and S100A8/A9 elevation. Therefore, targeting inherently immunosuppressive MDSC may be exploited for efficacious treatment of T‐cell driven autoimmune disorders such as MS.

## CONFLICT OF INTEREST

The authors declare no potential conflicts of interest with respect to the research, authorship and/or publication of this article.

## AUTHOR CONTRIBUTION

**Zhongkun Zhongkun Wang:** Formal analysis (lead); Software (equal); Writing‐original draft (equal). **Ge Zheng:** Formal analysis (equal); Resources (equal). **Guangjian Li:** Resources (equal). **Mengkun Wang:** Resources (equal); Software (equal). **Zhanchuan Ma:** Software (equal); Writing‐original draft (equal). **Huimin Li:** Software (equal). **Xiang‐Yang Wang:** Supervision (equal). **Huanfa Yi:** Conceptualization (lead); Funding acquisition (lead); Project administration (lead); Supervision (lead).

## Supporting information

Fig S1

## Data Availability

The data used to support the findings of this study are available from the corresponding author upon request.
