# Supplementary material for: Methylprednisolone alleviates multiple sclerosis by expanding myeloid‐derived suppressor cells via glucocorticoid receptor β and S100A8/9 up‐regulation
Source: J Cell Mol Med. 2020 Oct 23;24(23):13703–14. doi: 10.1111/jcmm.15928 (PMC7753844; doi:10.1111/jcmm.15928)

Supplementary figure. Suppressive function of MDSCs and their subsets from splenocytes were evaluated between the EAE and MPPT groups on the 16^th^ day.


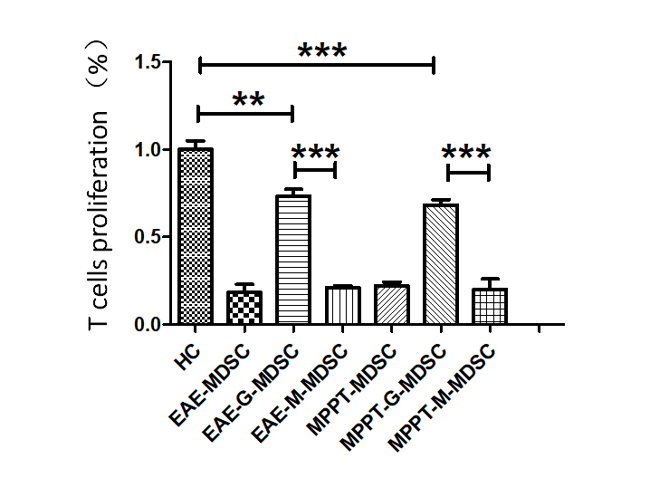

Supplement: Supplementary file 1 — Fig S1 [file JCMM-24-13703-s001.docx]
